# Feasibility and usefulness of a leadership intervention to implement evidence-based falls prevention practices in residential care in Canada

**DOI:** 10.1186/s40814-019-0485-7

**Published:** 2019-08-19

**Authors:** Wendy Gifford, Krystina B. Lewis, Ann Catrine Eldh, Val Fiset, Tara Abdul-Fatah, Anna Cristina Aberg, Kednapa Thavorn, Ian D. Graham, Lars Wallin

**Affiliations:** 1 Center for Research on Health and Nursing, University of Ottawa, 451 Smyth Road, Ottawa, Ontario Canada; 2 Faculty of Health Sciences, School of Nursing, University of Ottawa, 451 Smyth Road, Ottawa, Ontario Canada; 3 Faculty of Medicine, Department of Medicine and Health, Linköping University, SE-581 83, Linköping, Sweden; 4 School of Education, Health and Social Studies, Dalarna University, Högskolegatan 2, Falun, Sweden; 5 Department of Public Health and Caring Sciences, Uppsala University, Uppsala, Sweden; 6 Clinical Epidemiology Program, Ottawa Hospital Research Institute, 501 Smyth Road, Ottawa, Ontario Canada; 7 School of Epidemiology and Public Health, University of Ottawa, 600 Peter Morand Crescent, Ottawa, Ontario Canada; 8 Centre for Practice-Changing Research, Ottawa Hospital Research Institute, 501 Smyth Road, Ottawa, Ontario Canada; 9 Department of Health Care Science, University of Gothenburg, Gothenburg, Sweden; 10 Department of Neurobiology, Care Sciences and Society, Karolinska Institute, Stockholm, Sweden

**Keywords:** Implementation leadership, Fall prevention, Evidence-based practice, Nursing, Residential care

## Abstract

**Background:**

Leadership is critical to supporting and facilitating the implementation of evidence-based practices in health care. Yet, little is known about how to develop leadership capacity for this purpose. The aims of this study were to explore the (1) feasibility of delivering a leadership intervention to promote implementation, (2) usefulness of the leadership intervention, and (3) participants’ engagement in leadership to implement evidence-based fall prevention practices in Canadian residential care.

**Methods:**

We conducted a mixed-method before-and-after feasibility study on two units in a Canadian residential care facility. The leadership intervention was based on the Ottawa model of implementation leadership (O-MILe) and consisted of two workshops and two individualized coaching sessions over 3 months to develop leadership capacity for implementing evidence-based fall prevention practices. Participants (*n* = 10) included both formal (e.g., managers) and informal (e.g., nurses and care aids leaders). Outcome measures were parameters of feasibility (e.g., number of eligible candidates who attended the workshops and coaching sessions) and usefulness of the leadership intervention (e.g., ratings, suggested modifications). We conducted semi-structured interviews guided by the Implementation Leadership Scale (ILS), a validated measure of 12-item in four subcategories (proactive, supportive, knowledgeable, and perseverant), to explore the leadership behaviors that participants used to implement fall prevention practices. We repeated the ILS in a focus group meeting to understand the collective leadership behaviors used by the intervention team. Barriers and facilitators to leading implementation were also explored.

**Results:**

Delivery of the leadership intervention was feasible. All participants (*n* = 10) attended the workshops and eight participated in at least one coaching session. Workshops and coaching were rated useful (≥ 3 on a 0–4 Likert scale where 4 = highly useful) by 71% and 86% of participants, respectively. Participants rated the O-MILe subcategories of supportive and perseverant leadership highest for individual leadership, whereas supportive and knowledgeable leadership were rated highest for team leadership.

**Conclusions:**

The leadership intervention was feasible to deliver, deemed useful by participants, and fostered engagement in implementation leadership activities. Study findings highlight the complexity of developing implementation leadership and modifications required to optimize impact. Future trials are now required to test the effectiveness of the leadership intervention on developing leadership for implementing evidence-based practices.

## Background

Evidence-based clinical and organizational decision-making is required to strengthen the quality of health care and improve patient outcomes [1]. Implementation science research highlights that evidence-based decision-making in clinical practice is largely dependent on contextual factors [2]. Leadership is one of those contextual factors and considered critical for creating a supportive environment and facilitating the implementation of research evidence into health care practices, known as evidence-based practices [3–7].

Research has shown that the leadership of both formal and informal leaders positively influences the implementation of evidence-based practice, while its absence is a barrier [9–12]. Formal leaders include managers that oversee staff, have budgetary accountabilities, and are responsible for the operations of a clinical unit [13]. In contrast, informal leaders include point of care staff who are perceived by their colleagues to be credible and influential for influencing change and evidence-based practice [14]. Little is known about how to build leadership capacity of formal and informal leaders to influence the implementation of evidence-based practices [8].

With an aging population, improving the implementation of evidence-based practice for common and costly geriatric syndromes such as falls are needed [15]. Falls are the leading cause of fatal and non-fatal injuries in adults aged 65 years or older and represent substantial financial and human costs such as pain, suffering, and reduced quality of life [15]. Despite the existence of strong research evidence on how to reduce falls and fall injuries in the elderly, researchers have largely attributed high fall rates in health care environments to a lack of providers implementing evidence-based care [16, 17]. Implementation of evidence-based practices to prevent falls is essential to decrease the incidence and injuries associated with falls. While multifaceted programs have demonstrated some degree of efficacy [18], other attempts suggest that implementation of evidence-based fall prevention practices remain elusive [19]. A recent implementation study of a nurse-led fall prevention program suggested that enablers to fall prevention included education, training, and improvements in implementation leadership [20]. We developed a theory-based implementation leadership intervention aimed at building leadership capacity of formal leaders and informal clinical leaders to encourage implementation of evidence-based practices in long-term residential care for the elderly [21]. While we intend to evaluate the impact of the leadership intervention in a larger trial, we first needed to assess the feasibility and perceived usefulness of the content and delivery within the organizational setting.

## Aims and objectives

The aims of this study were to explore the feasibility of delivering and the perceived usefulness of a theory-based leadership intervention to influence the implementation of evidence-based fall prevention practices in a Canadian residential care setting (henceforth referred to as the “leadership intervention”). Specific objectives were to explore (1) feasibility of delivering the leadership intervention including intervention costs, (2) usefulness of the leadership intervention to participants (i.e., health care managers and clinical leaders), and (3) engagement in the leadership behaviors targeted in the intervention.

## Methods

A mixed-method, before-and-after feasibility study, was conducted in one Canadian residential care facility. The University of Ottawa Research Ethics Board (H02-16-14) and the participating organizational Research Ethics Board provided ethical approval for the study. We obtained written informed consent from all participants.

### Setting and participants

Study activities occurred on two units of a 7-unit 198-bed bilingual (French and English) residential care facility in Ottawa, Canada. The facility employed approximately 65 registered nursing staff and 150 unregulated care aids. Designated as a long-term care Best Practice Spotlight Organization in 2015 by the Registered Nurses Association of Ontario (RNAO), the facility was committed to implementing RNAO evidence-based guidelines to improve patient care [22]. Senior hospital administrators identified fall prevention as a priority clinical topic to address through the implementation of evidence-based care.

We purposefully recruited formal and informal leaders on the two participating units. Formal leaders were managers and a clinical educator responsible for training and supervising clinical staff. Informal leaders included staff nurses and unregulated care aids who had been identified by their managers as influential in practice with their peers. Each unit was composed of one Registered Nurse manager, one Registered Practical Nurse, and one to six unregulated health care aids per unit depending on the shift (days = 3–6, evenings = 2–4, nights = 1–2). The educator worked days for all the units in the facility. Together, the two participating units had a total of 48 residents pre-intervention and 46 post-intervention.

Recognizing the importance of senior leaders’ commitment to evidence-based practice, the study principal investigator (WG) held numerous meetings with two senior leaders (director of patient care and director of professional practice) to tailor the intervention strategy to the organizational context. Similar to an integrated knowledge translation approach where researchers work with knowledge users to co-develop research [23], the researcher worked with senior leaders to determine the most feasible and practical way to deliver the intervention. During the meetings, we reviewed the number of falls in the organization and the leadership knowledge and skills that leaders require to implement evidence-based practices, for example, prioritizing the evidence-based practice recommendations, setting goals and planning, providing support, and communicating clear and consistent messages. Senior leaders confirmed the need for both formal and informal leaders to develop implementation leadership knowledge and skills. They also reported that the intervention aligned with other quality improvement initiatives in the organization such as improving toileting routines for residents.

### The leadership intervention

The purpose of the leadership intervention was for participants to develop implementation leadership knowledge, skills, and interpersonal processes (i.e., behaviors) to influence staff’s use of evidence-based fall prevention practices. Guided by the principles of the UK Medical Research Council Complex Interventions Framework [24], the leadership intervention was informed by the Ottawa Model of Implementation Leadership (O-MILe) [21, 25, 26]. The O-MILe is a theoretical model based on leadership theory and empirical research that identifies the knowledge, skills, and interpersonal processes to facilitate the implementation of evidence-based practices. The embedded mechanisms in the O-MILe are that successful implementation requires leaders to understand site-specific evidence-practice gaps, implementation strategies, and how leadership influences planned change processes. Leaders must also have the skills to prioritize change, set target goals, and facilitate staff to practice evidence-based care for improved outcomes [21].

The O-MILe explicates the relations, change, and task-oriented leadership behaviors to facilitate the implementation of evidence-based practices [21]. Relation-oriented behaviors include supporting, developing skills, and recognizing others to increase cooperation and commitment. Change-oriented behaviors are concerned with a commitment to support change and creating a sense of need. Task-oriented behaviors include planning, clarifying roles, monitoring, and efficiently using resources [21]. The behavioral categories of relations, change, and task-oriented leadership have a strong empirical basis in leadership effectiveness [27, 28].

**Fig. 1:**
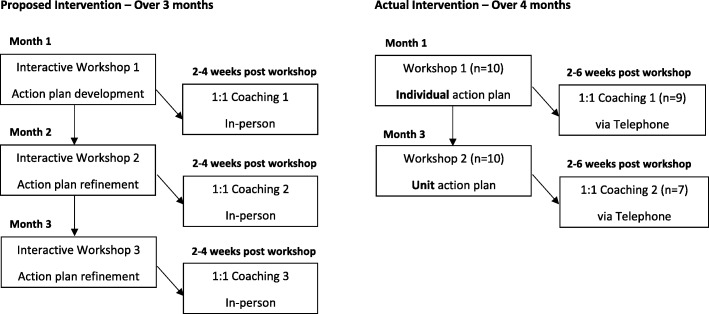
Flow chart of Intervention Components

The leadership intervention was designed to be delivered over 3 months. It included three interactive workshops and three individualized coaching sessions, both of which have shown to be effective strategies for developing leadership to address workplace challenges [29–31] (see Fig. 1 for components of the intervention).

#### Workshops

The number and duration of the workshops were adapted from the original study plan after meeting with senior administrators to make it easier for hospital administrators to find replacement staff for study participants to attend. Thus, two 3-h workshops were held (instead of three 2-h workshops).

The workshops were conducted in English and French by a fluently bilingual PhD candidate Registered Nurse (VF) and the principal investigator (WG). Content included leadership theory, evidence-based fall prevention practices, and implementation strategies. During the workshops, participants identified fall prevention practices to focus the implementation, set target goals for change, and develop leadership action plans.

Workshop participants prioritized six evidence-based practices for implementation that were compatible with other organizational directions, relatively easy to implement, and observable during the intervention period. Four of the practices came from the RNAO best practices guideline *Prevention of Falls and Falls Injuries in the Older Adult* [32], and two from RNAO guidelines *Promoting Continence Using Prompted Voiding* [33] and *Prevention of Constipation in the Older Adult Population* [34] with implications for fall prevention. The six chosen practices were from the 19 practices outlined in the institution’s standardized care plan. The remaining 13 practices would be the focus of implementation in the future.

After identifying the practices for implementation, participants set target goals that represented the percentage of residents’ charts they would see the evidence-based practices documented in. The practices and the target goals for implementation were (1) educating clients and families about fall prevention (goal 50%); (2) identifying and modifying equipment in the environment that increases risks for falls and fall injuries, in this case, marking the optimal bed height for each resident on the wall (goal 80%); (3) developing an interdisciplinary exercise plan through physiotherapy consult (goal 75%); (4) establishing a toileting plan (goal 50%); (5) increasing fluid intake (goal 50%); and (6) increasing dietary fiber (goal 30%).

In the workshops, participant developed a leadership action plan that identified the relations, change, and task-oriented leadership behaviors they would engage in to implement the fall prevention practices [21]. Each participant developed an individual leadership action plan in the first workshop that specified the leadership behaviors they would individually engage in. Whereas in the second workshop, participants developed a unit-level team leadership action plan of the leadership behaviors they would collectively engage in as a team to implement fall prevention practices.

#### Coaching

Telephone coaching sessions of 15–30 min per participant were provided by the workshop facilitators (VF, WG) 2 to 6 weeks after each workshop. During coaching, participants discussed the implementation leadership behaviors identified in their action plans, factors that promoted or hindered operationalizing them, and modifications to their leadership action plans as required. The coaching facilitators updated the action plans based on these discussions and emailed the revised version back to participants.

### Outcomes and measures

The primary outcomes were parameters of feasibility and usefulness of the intervention. Feasibility outcomes included (a) number of eligible candidates who attended workshops, (b) proportion of workshop participants who initiated a written leadership action plan, (c) number of workshop participants who attended the coaching sessions, (d) costs of designing and delivering the intervention, and (e) ability to collect data on fall rates, severity of injuries from falls, and documented evidence-based practices identified in the workshop. The usefulness of the intervention was based on ratings of 3 and above on a 5-point Likert scale, where 0 = not at all useful and 4 = extremely useful for developing implementation leadership behaviors.

Secondary outcomes were participants’ perceptions of practicing the implementation leadership behaviors developed within the intervention and barriers and facilitators to practicing them. We explored the leadership behaviors through ratings of the Implementation Leadership Scale (ILS) [35] and qualitative descriptions. The ILS is a 12-item measure of unit-level leadership for implementing evidence-based practice with four subscales: proactive, knowledgeable, supportive, and perseverant leadership [35]. Participants rate the extent to which they engage in the leadership behaviors represented in the ILS on a 5-point Likert scale (0 = not at all, 4 = very great extent), with higher ILS scores indicating stronger leadership for implementing evidence-based practices. Previous research has demonstrated a Cronbach’s alpha coefficient of 0.98 on total ILS scores for internal consistency and has shown good convergent validity and discriminant validity on the total scale and all four subscales [35]. The ILS conceptually maps to the O-MILe, demonstrating compatibility as a measure for an O-MILe-based intervention [21].

### Data collection

We collected feasibility data related to recruitment, engagement of participants, and costs through a process log during intervention delivery. Costing data were classified as operating costs (production of educational materials, workshop preparation and delivery, tele-communication, office supplies) and staff participation costs (based on mean hourly wages). Data on fall rates and severity of injuries from falls were collected from the organization’s electronic administrative database 3 months pre-intervention and 3 months post-intervention. We also conducted a 3-month pre/post-chart audit on staff’s documentation of the six evidence-based fall prevention practices selected for implementation. Chart audit data was extracted from the electronic health records that had been newly installed just prior to the pre-intervention data collection period.

We conducted semi-structured interviews after the final coaching sessions to explore the usefulness of the leadership intervention and the leadership behaviors. Interviews were conducted by a bilingual PhD candidate (KL) experienced in qualitative interviewing techniques who was not involved in the intervention delivery. The interview guide was based on the ILS. Participants were first asked to rate the extent to which they engaged in each of the ILS leadership behaviors. We then asked participants to expand on their ILS ratings and provide examples of what they did to support the implementation of fall prevention practices. For example, after rating the extent to which they developed a plan for implementation, participants were asked to give examples of what was in the plan and what they did to operationalize the plan. We probed for the barriers and facilitators to practicing the leadership behaviors targeted in the leadership intervention, in addition to suggestions for improving the intervention content and delivery.

Three months after the interviews, a focus group discussion took place where participants were presented with the qualitative study findings and asked to further discuss their implementation leadership behaviors. Participants then completed the ILS to reflect how the unit level team collectively engaged in the leadership behaviors for each ILS item (referred to as team ILS). The focus group discussion was facilitated by the same bilingual PhD candidate who conducted the intervention workshops.

### Data analysis

We summarized feasibility outcomes using frequencies and proportions, describing continuous outcomes with means and ranges. Cost data were summarized using mean and standard deviation (SD) and presented in 2018 Canadian dollars. Staff participation costs were calculated by multiplying the mean hourly wages with the number of hours that staff participated in the workshop and coaching sessions. Miscellaneous costs included transportation expenses for research staff to collect chart audit data for the evaluation of the fall prevention strategies and to attend the workshop.

Semi-structured interviews were audiotaped and transcribed verbatim and entered into NVIVO 10 qualitative software. Qualitative data were deductively coded into categories that corresponded to ILS subscales (i.e., proactive leadership, knowledgeable leadership, supportive leadership, and perseverant leadership). Two researchers inductively analyzed the data into themes for each category (KL, TAF) [36]. We held investigator group meetings during the analysis to iteratively discuss findings as they emerged. At the focus group meeting, we presented results to study participants who commented on and confirmed findings (member checking). We took field notes during the focus group meeting and incorporated the data into findings.

As described by the ILS tool developers to calculate scores for the ILS, we first computed a mean score for each set of three items within a given subscale [35]. We report the median rating for the subscale and range of computed mean scores for each subscale to accommodate for the small sample size [35]. We categorized barriers and facilitators to engaging in the leadership behaviors by ILS subscale (i.e., proactive, knowledgeable, supportive, and perseverant leadership) [35].

## Results

Pre-intervention data was collected between October and December 2016. The intervention was delivered between January and April 2017. Post-intervention data was collected between May and July 2017.

### Participant characteristics

Workshop participants (*n* = 10, 100% response rate) included formal leaders (*n* = 3, unit managers and clinical educator) and informal leaders (*n* = 7, nurses and unregulated care aids) (*n* = 3). Workshop participants had been employed at the organization for a median of 10 years (range 2–17) and held their current position for a median of 4 years (range 1–10). Post-intervention, seven workshop participants engaged in an individual interview and six interview participants attended the focus group (Table 1).

**Table 1:** Participant characteristics

	Workshop participants (*n* = 10)	Interview participants (*n* = 7)	Focus group participants (*n* = 6)
Position, *n* (%)			
Formal leaders (unit manager/educator)	3 (30%)	3 (43%)	2 (33%)
Informal leaders (RNs, care aids)	7 (70%)	4 (57%)	4 (67%)
Years employed at organization (mean, range) ^*^	10 (2–17)	10 (2–17)	11 (3–17)
Years employed in current position (mean, range)	4 (1–11)	5.7 (1–11)	5.8 (1–11)
Highest education level obtained			
High school/college diploma	3^*^ (43%)	3 (57%)	3 (50%)
Undergraduate/graduate degree	4* (57%)	4 (43%)	3 (50%)

*Seven participants responded

### Feasibility outcomes

#### Attendance—workshops and coaching sessions

All eligible participants who were invited to the workshops attended (*n* = 10). One clinical leader withdrew from the study after the second workshop citing heavy workload and thus did not complete the second coaching session or participate in post-intervention data collection. For the subsequent two coaching sessions, eight and seven participants engaged respectively. Scheduling conflicts were given as why participants were not able to participate in coaching sessions.

#### Leadership action plans

All participants began writing an individualized leadership action plan during the first workshop to facilitate implementation of the six identified practices. No participants were able to complete their action plans during the first workshop due to time restraints. In the second workshop, facilitators gave participants the choice to continue developing an individualized leadership action plan or develop a unit-level team leadership action plan to document how they would collectively lead implementation. Participants chose to develop a team leadership action plan, stating they would achieve greater success through a coordinated and cohesive team plan rather than an individualized approach. At the end of the second workshop, a team leadership action plan was completed for each participating unit.

### Ability to collect data

#### Falls

Standardized data on fall rates were not available, nor were data on severity of injuries from falls. Data on the total number of falls were available and collected from the organization’s electronic database, showing 22 falls in 48 residents in 3 months pre-intervention and 30 falls in 46 residents in 3 months post-intervention. It was not clear from the data collected how many residents had fallen from the total number of falls.

#### Documentation of evidence-based practices

All data on the fall prevention strategies identified for implementation were documented and available through the electronic charts with the exception of physiotherapy referral, which was not available through the electronic charting system pre-intervention but was available post-intervention (Table 2). Chart audit data showed that none of the six evidence-based practices participants selected for implementation had been documented as completed on all residents. We noted an increase in the documentation of *client and family education* pre/post-intervention, while two practices (*encourage adequate fluid intake and dietary fiber*) had a small decrease in documentation pre/post intervention.

**Table 2:** Documentation of evidence-based practices pre/post-intervention

Evidence-based practice identified for implementation	Level of evidence*	Pre-intervention (%)	Post-intervention (%)
Provide client and family education^∞^	IV	2	35
Identify and modify equipment/environment^∞^ (i.e., mark optimal bed height on wall)	Ia	0	4
Referral to physiotherapy for exercise plan^∞^	Ib	Unavailable	46
Develop toileting plan^∞^	IV	2	4
Encourage adequate fluid intake^#^	III	39	35
Encourage dietary fiber^◊^	III	18	15

^*^Higher levels of evidence suggest fewer sources of bias. For example, level Ia is evidence from systematic reviews of randomized controlled trials, whereas level IV is from non-experimental observational studies, such as descriptive and/or qualitative studies

^∞^Registered Nurses of Ontario (2011) guideline: *Prevention of Falls and Falls Injuries in the Older Adult* [32]

^#^Registered Nurses of Ontario (2011) guideline: *Promoting Continence Using Prompted Voiding* [33]

^◊^Registered Nurses of Ontario (2011) guideline: *Prevention of Constipation in the Older Adult Population* [34]

### Costs of designing and delivering the intervention

We estimated the total costs associated with developing and delivering the intervention at C$6100 (C$610 per individual). Operating costs were the main cost driver (C$3200) accounting for 52.5% of the total intervention costs. Staff participation costs was estimated to be C$2500, while transportation expenses for research staff to collect chart audit data for the evaluation of the fall prevention strategies and to attend the workshop were C$400.

### Usefulness of the intervention

Out of the seven participants who rated the intervention for usefulness to develop implementation leadership skills (Likert scale of 0–4, where 0 = not at all useful and 4 = highly useful), six rated the workshops and coaching sessions > 3 (range = 2–4, median = 3).

#### Suggested modifications to the intervention

Participants suggested ways to improve the content and delivery of the intervention. For the workshops, participants suggested enhancing the curricula to include ways to adapt evidence-based clinical practices to the organizational context, in this case, fall prevention practices in residential care facilities. They also suggested role-playing exercises to provide opportunities to practice leadership behaviors in a safe environment. Logistically, some participants stated the 3-h workshop was difficult when combined with their work-shift (despite being replaced at work to attend the workshops), while others expressed the need for more frequent team-based sessions to review their leadership action plans and activities, discuss challenges, and modify their action plans as required.

Participants appreciated the opportunity to reflect on their leadership practices in the coaching sessions. However, they suggested coaching should occur face-to-face in the clinical setting and in close proximity to the workshop dates to allow easy recall of workshop content. They also believed that more staff should have the opportunity to participate in implementation leadership development, so the units could develop a critical mass of peer leaders for evidence-based practice. Intervention participants suggested that staff who self-identify as clinical leaders should also be able to participate rather than relying on senior administrators’ decisions about which units and staff should participate.

### Leadership behaviors post-intervention

#### Implementation Leadership Scale

Computed *Implementation Leadership Scale (*ILS) subscale scores for participant (*n* = 7) ratings of individualized implementation leadership were highest for supportive and perseverant leadership, with the median computed scores of four on a 5-point Likert scale where 0 = not at all and 4 = very great extent (higher scores indicate stronger implementation leadership). For the team ILS, participants (*n* = 6) highest computed subscale scores were knowledgeable leadership (median = 4) and supportive leadership (median = 4) (Table 3).

**Table 3:** Leadership intervention participants’ ratings of the Implementation Leadership Scale (ILS) scores (supervisor version)

Scale items	Individual ratings (*n* = 7) Median (range of mean scores)	Team ratings (*n* = 6) Median (range of mean scores)
1. Proactive leadership		
- Developed a plan to facilitate implementation of evidence-based fall prevention practices	3 (2–4)	3 (2–3)
- Removed obstacles to implement evidence-based fall prevention practices	3 (2–4)	4 (2–4)
- Established clear standards for implementing evidence-based fall prevention practices	2 (1–4)	3 (2–4)
Subscale total	3 (2–3)	3 (3–4)
2. Knowledgeable leadership		
- Is knowledgeable about evidence-based fall prevention practices	3 (3–4)	4 (3–4)
- Is able to answer staff’s questions about evidence-based fall prevention practices	3 (2–4)	3 (3–4)
- Knows what he/she is taking about when it comes to evidence-based fall prevention practices	4 (3–4)	4 (3–4)
Subscale total	3 (3–4)	4 (3–4)
3. Supportive leadership		
- Recognized employee efforts toward implementation of evidence-based fall prevention	4 (3–4)	4 (4)
- Supported employee efforts to learn more about evidence-based fall prevention practices	3 (3–4)	4 (3–4)
- Supported employee efforts to use evidence-based fall prevention practices	4 (3–4)	3.5 (3–4)
Subscale total	4 (3–4)	4 (3.5–4)
4. Perseverant leadership		
- Persevered through ups and downs of implementing evidence-based fall prevention	4 (3–4)	3 (2–4)
- Carried on through the challenges of implementing evidence-based fall prevention practices	4 (3–4)	3 (3–4)
- Reacted to critical issues regarding implementation of evidence-based fall prevention practices	3 (3–4)	3.5 (3–4)
Subscale total	4 (3–4)	3 (3–3.5)
Total score	3 (2–4)	3.5 (3–4)

#### Barriers and facilitators to leading implementation

Barriers and facilitators to leading implementation are shown in Table 4. Participants perceived barriers to enacting leadership in all ILS subscale categories. Barriers included unclear implementation roles, lack of authority to make change, inconsistent messages and commitment from senior leaders, and difficulties in planning as a team. Facilitators to leading implementation included active listening, engaging and encouraging feedback from staff, recognizing and supporting staff, and emphasizing residents’ safety.

**Table 4:** Barriers and facilitators to leading implementation of evidence-based fall prevention practices

ILS subscale category (35)	Barriers	Facilitators
Proactive leadership	• Unclear implementation roles • Lack of authority to facilitate change • Difficulty planning as a team • Lack of process to monitor goals and outcomes • Lack of resources for new initiatives (i.e., staff and time)	• Team coordination to develop implementation plan and strategy • Link implementation to other organizational initiatives
Knowledgeable leadership	• Inconsistent messages from senior leaders	• Emphasize residents’ safety as reason for implementation
Supportive leadership	• Inability to engage staff • Limited communication with staff and leadership team	• Active listening • Engage and encourage feedback from staff • Recognize and support staff efforts and contributions
Perseverant leadership	• Lack of commitment	• Commitment

## Discussion

Falls amongst seniors living in residential care facilities in Canada have serious effects on their health including debilitating injuries and early death [15]. Evidence-based fall prevention strategies exist to reduce falls and falls’ injuries; however, implementation remains a formidable challenge and staff in residential care facilities inconsistently and ineffectively apply these strategies [16, 17]. Recognizing the importance of leadership for implementing evidence-based practices, the primary objective of this study was to field test an implementation leadership intervention aimed at improving fall prevention practices in residential care. Findings indicate that aspects of the leadership intervention were feasible to deliver when adapted to the organization’s staffing context and considered useful to participants for developing implementation leadership knowledge and skills.

### Feasibility and usefulness of the leadership intervention

With only one intervention participant (10%) withdrawing from the intervention, this study showed that rates of recruitment and retention were achievable for a larger study. Researchers conducted two 3-h workshops after careful negotiations with senior administrators to tailor the frequency and length of the sessions to the logistical and practical needs of the organization. Researchers have described consensus-based negotiations with decision makers as promising ways to develop and deliver feasible and relevant interventions with the potential for high impact [37].

All coaching sessions occurred by telephone and participants suggested they would be more effective if they occurred in the clinical setting. Despite this, the majority of participants (86%) considered the coaching sessions useful. Both group and individualized coaching have been shown to positively impact nurses’ leadership skills when delivered as part of a structured leadership development program [29, 38]. A systematic review of 52 articles demonstrated coaching was effective in leadership development; however, gaps remain in understanding how coaching should be delivered for the most positive effects [30]. Findings from field testing the intervention indicates that negotiations with decision-makers of participating organizations should include the format and location of the coaching sessions, along with the frequency and timing in relation to the workshop dates.

Some of the outcome data we planned to collect in this study were not available at the participating organization, specifically the type and severity of injuries from falls. While fall rates are an essential reporting requirement for residential care homes in Canada [39], the reporting of other related data points varies between organizations. With a complexity of factors impacting health outcomes for older people in residential care homes such as cognitive impairment, polypharmacy, vertigo and previous falls (to name a few) [40, 41], researchers must carefully consider the type of data available from participating organizations when selecting outcomes including what constitutes a fall, fall injury, and severity of injury. Researchers can then identify conceptually relevant outcomes that are feasible to collect and meaningful to participants that can be benchmarked and compared to previous and future studies.

It was interesting to note that the number of falls increased after the intervention. Given the purpose of the study was to explore the feasibility of delivering the leadership intervention and the usefulness of the intervention to develop implementation leadership behaviors, the study was not powered to detect causal differences in fall prevention practices or fall rates. The sample size was small and the leadership intervention was not delivered as planned with fewer workshops and coaching sessions than intended. In addition, we collected data on the absolute number of falls which is not a standardized method of reporting fall rates and may not be a clinically significant measure of understanding fall prevention strategies. Rather, the rate of falls per person year or per 1000 occupied patient days is a standardized reporting method and should be collected to allow comparisons to other studies and examine trends over time [42–44]. As a complex intervention with multiple components, future leadership intervention studies should ensure an adequate sample size and capture the intervention fidelity to understand what works in different contexts [45].

### Leadership behaviors

Our findings showed that, following the leadership intervention, participants scored highly on the ILS suggesting they perceived they engaged in considerable implementation leadership behaviors. Specifically, participants stated they recognized and appreciated employee efforts, continued implementing fall prevention strategies despite challenges, and had increased knowledge about evidence-based fall prevention practices. Yet, despite favorable findings, we did not observe any change in staff’s documentation of the fall prevention practices they had intended to change. We propose three possible explanations. First, this could have been the result of insufficient dose of the leadership intervention. Second, participants may have required more time to develop their leadership capacity to influence staff. Third, low fidelity of the coaching sessions may have contributed to participants’ lack of influence on fall prevention practices. Consistent with this feasibility study, a previous pilot study in Sweden similarly reported an implementation leadership intervention was perceived to be beneficial to managers; however, limited impact was observed in changing clinical practices with rehabilitation therapists [46]. A meta-analysis showed that overall, effectiveness of leadership development programs varies greatly with some programs tremendously effective and others not at all [31]. For example, the effect size for knowledge outcomes ranged from .96 to 1.37, expertise outcomes from .35 to 1.01, and system outcomes averaging .39 [31]. Our study findings suggest there is a need to focus on applying implementation leadership practices in work settings to influence evidence-based practices by clinical staff.

The leadership intervention utilized the O-MILe as the theoretical foundation for developing leadership capacity to influence the implementation of evidence-based practices. Consistent with skill development approaches to leadership development [47, 48], the O-MILe focuses on leaders’ knowledge and skills to be visionary about change, proactive in setting goals in their work environments, and engaged with staff while securing necessary resources for the provision of effective high-quality care. Theory on leadership development suggests that individual’s progress from novice, to intermediate, to expert leadership levels and at each stage, different knowledge, and information-processing capabilities can be acquired as people interact with their environments [47, 48]. Adult learning principles further suggest that people react differently to training based on their individual needs, learning styles, and preferences [49]. A meta-analysis showed that improvements in both leadership knowledge and practical skills can be made if sufficient front-end analysis is done to offer the right content and format to the right leaders in leadership development programs [31]. As suggested by participants in our study, ongoing self-assessments is an important component of leadership development [31].

Our findings support the notion of a team-based model of implementation leadership where different types of leadership expertise are needed to fill different leadership roles. Scholars have increasingly emphasized leadership as a team process that enables staff to navigate the complexities of their environment to meet organizational goals [50]. As such, implementation leadership may be thought of as a social process enacted by a team to mobilize evidence-based practices, rather than an individually driven dyadic process between a leader and followers. Researchers in Sweden suggested that implementation interventions that engage both formal and informal leaders are needed, and interventions in seniors’ homes that focus specifically on front-line managers had limited impact on developing leadership behaviors for implementing evidence-based practices [51]. While research on team-focused leadership development is still in its infancy [50], our study participants confirmed that team leadership that includes both formal and informal leaders was important for implementing evidence-based practices. Team leadership development tailors the developmental needs of each member to the shared collective needs of the team, whereby the team assumes the responsibility and leadership structure for implementing evidence-based practice equally.

### Strengths and limitations

There are limitations to this study. Some of our outcomes relied on participant self-report, and we did not assess staff ratings of their leaders’ behaviors. We focused on the feasibility and usefulness of the intervention, and participants provided assessments of how much they were instrumentally applying the knowledge and skills they learned in the intervention including directions for further testing. Regarding limitations to the leadership intervention itself, participants were not able to complete their leadership action plans as planned in the first workshop, suggesting the need for more time to cover the intervention content and facilitate implementation leadership development. Assessment of feasibility and usefulness did not focus on specific strategies that were part of leadership action plans, and future studies should more clearly identify and assess such strategies. Finally, the intervention was only 3 months in length and we did not observe any changes in implementation of the evidence-based practices. This finding, together with the qualitative results, suggests that the leadership intervention needs a longer time to achieve desired effects.

We also note a number of study strengths. The leadership intervention is built on an empirically developed and theoretically supported approach to leadership development for implementing evidence-based practices [21]. Our process to determine how to deliver the intervention involved engagement with senior organizational leaders to tailor the strategy to the logistical context of the practice environment. We collected both quantitative and qualitative data, and participants included both formal and informal leaders who discussed barriers and facilitators to performing implementation leadership behaviors rather than barriers and facilitators to evidence-based practice as frequently reported in the literature [52, 53]. Finally, the study took place in a residential long-term care setting, an underrepresented and particularly challenging research setting [54].

## Conclusion

The leadership intervention was feasible to deliver, deemed useful by participants, and fostered reporting of implementation leadership activities in a residential care home in Canada. After the intervention, participants developed an action plan, were visionary about change, and engaged with staff to lead the implementation of evidence-based fall prevention practices. They also revealed barriers to leading implementation that included unclear role boundaries and challenges bringing the leadership team together. Although leadership in general has been shown to support implementation [7, 8], our intervention highlights the complexity of developing implementation leadership. Future trials are now required to test the effectiveness of the leadership intervention on developing leadership for implementing evidence-based practices.

## Data Availability

The datasets used during the current study are available from the corresponding author on reasonable request.
